# Primary Aldosteronism: Evaluation and Management in the Hypertensive Patient

**DOI:** 10.1007/s11906-026-01399-y

**Published:** 2026-11-03

**Authors:** Maria Balhara, Lily Owei, John Demko, Heather Wachtel, Jordana B. Cohen

**Affiliations:** 1 Perelman School of Medicine, Renal-Electrolyte and Hypertension Division, University of Pennsylvania, Philadelphia, PA USA; 2 Department of Surgery, Perelman School of Medicine, University of Pennsylvania, Philadelphia, PA USA; 3 Department of Biostatistics, Epidemiology, and Informatics, Perelman School of Medicine, University of Pennsylvania, Philadelphia, PA USA

**Keywords:** Primary aldosteronism, Screening, Hypertension, Blood pressure, Mineralocorticoid receptor antagonist, Adrenalectomy

## Abstract

**Purposeof Review:**

Primary aldosteronism (PA) is common, underdiagnosed, and associated with excess cardiorenal risk. We review recent changes in screening, diagnosis, treatment, and implementation.

**Recent Findings:**

Contemporary guidelines have broadened screening and reduced barriers to aldosterone-to-renin ratio testing through medication-tolerant, assay-aware interpretation. Confirmatory aldosterone suppression testing is increasingly de-emphasized, with selective use or omission in high-probability patients when results are unlikely to change management. Adrenal vein sampling remains standard for most surgical candidates, while molecular imaging is emerging as a noninvasive alternative. Adrenalectomy is preferred for lateralizing disease; mineralocorticoid receptor antagonists remain first-line therapy for bilateral or nonoperative disease, with renin response increasingly used to assess dosing adequacy. Finerenone and aldosterone synthase inhibitors are potential emerging options.

**Summary:**

PA care is shifting from a rigid multistep workup toward earlier detection and treatment-directed evaluation. Future work should define scalable screening pathways and compare outcomes of emerging diagnostic and therapeutic strategies.

## Introduction

Primary aldosteronism (PA) is a major and underrecognized contributor to the global burden of hypertension. PA is estimated to affect approximately 5% to 10% of all individuals with hypertension and over 20% of those with resistant or difficult-to-control hypertension, corresponding to more than 100 million affected individuals worldwide [1–3]. Despite its prevalence, PA is markedly underdiagnosed and therefore underrepresented in administrative and healthcare utilization data as well as clinical trials. This under recognition is clinically consequential, as PA is associated with substantially increased risks of cardiovascular and kidney morbidity and mortality, including stroke, atrial fibrillation, heart failure, and chronic kidney disease compared with primary hypertension. These risks are disproportionate to blood pressure elevation alone [4, 5]. A nationwide population-based study found 23% higher all-cause and 57% higher cardiovascular mortality among patients with PA compared with matched population controls, with the highest mortality risk among patients with untreated PA (hazard ratio 2.51, 95% confidence interval 1.72–3.67) [6].

PA is etiologically heterogeneous, most commonly reflecting bilateral aldosterone overproduction or lateralizing aldosterone-producing lesions, with rare familial forms accounting for a small proportion of cases [7]. PA also occurs across a spectrum of blood pressure severity. In a cross-sectional, observational study, the adjusted prevalence increased from approximately 11% in normotensive individuals to 16% in individuals with stage 1 hypertension, and 22% in those with stage 2 or resistant hypertension [3]. Growing evidence indicates that renin-independent aldosterone production exists along a continuum, with increased cardiovascular risk detectable even in individuals who do not meet conventional diagnostic criteria [8]. Therefore, earlier screening for PA, even among individuals without classical risk factors such as hypokalemia and resistant hypertension, may be warranted.

In this review, we examine recent advances in PA screening, diagnostic evaluation, subtype classification, and treatment, with particular emphasis on how evolving evidence and guidelines are shifting care toward broader detection, more pragmatic testing, and treatment-directed evaluation.

## Assessment of Patients with Primary Aldosteronism

### Who (and Why) to Screen

**Fig. 1:**
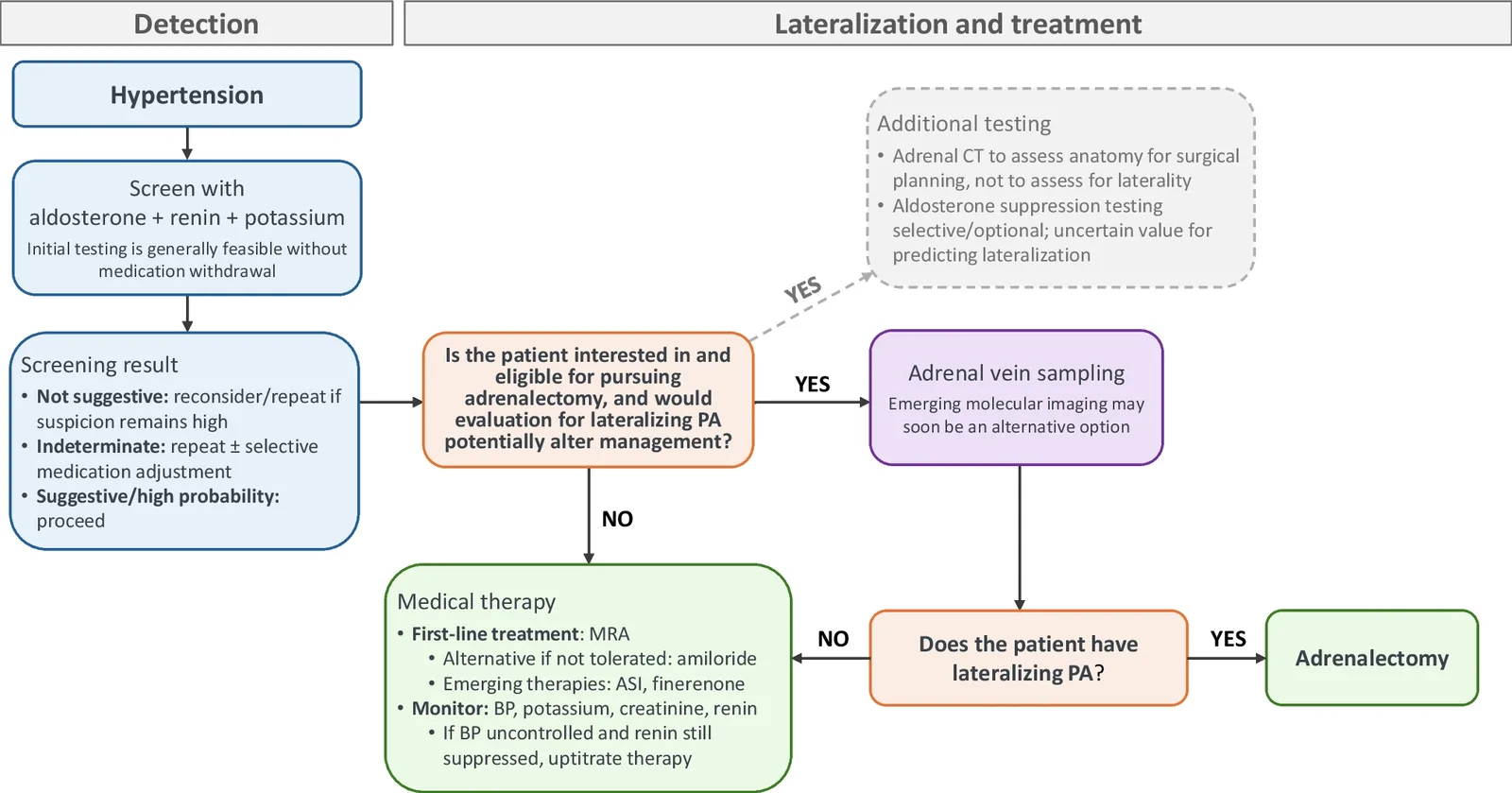
Contemporary Approach to the Evaluation and Management of Primary Aldosteronism. A positive PA screen generally consists of suppressed renin (PRA ≤ 1 ng/mL/h or DRC ≤ 8.2 mU/L), aldosterone ≥ 10 ng/dL by immunoassay or ≥ 7.5 ng/dL by LC–MS/MS, and an elevated ARR (> 20 for aldosterone [ng/dL]/PRA [ng/mL/h] or > 70 for aldosterone [pmol/L]/DRC [mU/L] with immunoassay; ARR thresholds are approximately 25% lower with LC–MS/MS). Results not meeting these criteria may warrant repeat testing when clinical suspicion is high or testing conditions are potentially confounded. Selected young patients with marked PA and a clear unilateral nodule may proceed to adrenalectomy without AVS. The dashed line and box indicate a selective or uncertain role. Abbreviations: ASI, aldosterone synthase inhibitor; CT, computed tomography; MRA, mineralocorticoid receptor antagonist; PA, primary aldosteronism

Current guidelines support broader screening compared with prior recommendations, which restricted testing to high-risk groups such as those with resistant hypertension or hypokalemia (Fig. 1). The 2025 Endocrine Society guideline recommends screening all individuals with hypertension, defined as BP ≥ 140/90 mm Hg. The 2024 European Society of Cardiology (ESC) and 2025 American Heart Association/American College of Cardiology (AHA/ACC) guidelines similarly recommend considering screening at this BP threshold, corresponding to stage 2 hypertension in the AHA/ACC classification [7, 9, 10]. Historically, screening for PA has been markedly underutilized, even among high-risk groups targeted by prior guidelines. A retrospective cohort study of 4660 adult patients with resistant hypertension, all of whom were eligible for PA screening, found that only 2.1% underwent screening within 2 years of meeting the criteria for resistant hypertension [11]. Similarly, screening rates among patients with hypertension and hypokalemia have been very low, ranging from 1.6% to 2.7% [12, 13]. In a broader population, Sivarajah et al. reported that although 43.8% of hypertensive patients met historical screening criteria, only 1.3% were tested [14]. Cohen et al. reported that among more than 200,000 U.S. Veterans with apparent treatment-resistant hypertension, 1.6% underwent screening for PA [15].

Although broader screening may facilitate earlier diagnosis and targeted treatment, direct evidence that near-universal screening improves long-term clinical outcomes remains limited. Furthermore, acknowledging that universal or near-universal screening may not be readily operationalized in many healthcare settings, pragmatic prioritization of groups with a high pretest probability of PA remains reasonable, including patients with resistant hypertension, stage 2 hypertension, hypokalemia, early-onset hypertension, adrenal incidentaloma, obstructive sleep apnea, or early stroke [7, 9, 10, 16, 17]. Risk-prediction approaches may help prioritize patients with a high pretest probability of PA [18]. However, importantly, health economic analyses support broader screening, with studies demonstrating favorable cost-effectiveness or cost savings across both high-risk and more general hypertensive populations [19–22].

### How to Screen

Screening for PA is performed using the aldosterone-to-renin ratio (ARR), which is calculated from the plasma aldosterone concentration and renin concentration measured in the outpatient setting. Renin may be measured as plasma renin activity or direct renin concentration, and aldosterone can be measured using immunoassays or liquid chromatography-tandem mass spectrometry. For initial screening, aldosterone, renin, and potassium should generally be measured in the morning with the patient seated and without dietary sodium restriction. Antihypertensive therapy does not need to be routinely withdrawn for initial screening. Results should be interpreted in the context of medication effects, with selective withdrawal and repeat testing when results are equivocal or clinical suspicion remains high. Mineralocorticoid receptor antagonists (MRAs), aldosterone synthase inhibitors, and ENaC inhibitors substantially increase renin can obscure renin suppression; when these agents cannot be safely discontinued, screening may still be informative, although interpretation can be more difficult. Borderline or discordant results should prompt repeated testing rather than exclusion of disease [7].

Even under simplified testing conditions, ARR interpretation remains complex because of medication effects, assay variability, and physiologic factors including age, kidney function, hormonal state, volume status, and sodium intake [23]. Immunoassays can substantially overestimate aldosterone relative to liquid chromatography-tandem mass spectrometry, particularly at lower concentrations [24]. These sources of variability make ARR interpretation context dependent and may contribute to underuse of screening.

## Diagnosis of Primary Aldosteronism

### Interpretation of Screening Results

Interpretation of screening results requires the integration of renin, aldosterone, ARR, and their relationships within the clinical context. A positive screen for PA is defined by the following three findings: suppressed renin, inappropriately elevated aldosterone, and an increased ARR. Guidelines provide thresholds for these components, although values vary by assay and measurement method. Suppressed renin (≤ 1 ng/mL/h as plasma renin activity or ≤ 8.2 mU/L as direct renin concentration), elevated aldosterone (≥ 10 ng/dL by immunoassay or ≥ 7.5 ng/dL by liquid chromatography-tandem mass spectrometry), and an elevated ARR are consistent with a positive screening result [7]. With immunoassay aldosterone, an ARR > 20 applies when aldosterone is expressed in ng/dL and PRA in ng/mL/h, whereas an ARR > 70 applies when aldosterone is expressed in pmol/L and DRC in mU/L; thresholds are approximately 25% lower with liquid chromatography-tandem mass spectrometry. Interpretation must also account for concurrent antihypertensive therapy. Renin-suppressing agents (e.g., β-blockers, central α_2_-agonists, and nonsteroidal anti-inflammatory drugs) may lead to false-positive results, whereas renin-stimulating agents (e.g., ACE inhibitors, angiotensin receptor blockers, diuretics) or medications that increase both renin and aldosterone (e.g., selective serotonin reuptake inhibitors) may result in false-negative findings [25, 26]. Hormonal contraceptives can also alter ARR in a formulation- and assay-dependent manner [7].

ARR interpretation is subject to substantial biological and analytic variability. Yozamp et al. reported coefficients of variation of approximately 31% for aldosterone and 45% for ARR, with more than 57% of patients with confirmed PA demonstrating at least one ARR below conventional screening thresholds [27]. Interpretation based on a single measurement is inherently variable and highly context dependent. Consequently, ARR results should be interpreted as probabilistic rather than definitive, and integrate clinical context, medication use, and repeated measurements when necessary. Repeat testing and context-specific laboratory interpretation may be particularly useful when results are borderline or discordant with the clinical phenotype. Referral to a clinician or center with expertise in PA may be particularly useful for patients with a positive screening test who may be candidates for surgery, persistently equivocal results despite high clinical suspicion, or testing conditions that complicate interpretation.

### Role of Aldosterone Suppression Testing

Confirmatory testing is traditionally used to establish a diagnosis of PA following an abnormal screening result. The principal confirmatory tests include saline infusion, oral sodium loading, captopril challenge, and fludrocortisone suppression. Recent guidelines increasingly support a shift from universal to selective use of confirmatory testing. The 2025 Endocrine Society guidelines recommend confirmatory aldosterone suppression testing primarily for patients with an intermediate probability of lateralizing PA who are willing and able to pursue surgical therapy [7]. In other settings, confirmatory testing may not be necessary.

However, emerging evidence raises questions about even this more selective role for suppression testing. A meta-analysis by Leung et al. demonstrated substantial heterogeneity in testing protocols, diagnostic thresholds, and reference standards, with a high risk of bias across studies and marked systematic overestimation of diagnostic accuracy [28]. In a separate prospective study, the seated saline suppression test demonstrated poor discrimination between treatment-response groups (area under the curve 0.62; 95% CI, 0.45–0.79) [29]. More recently, a blinded clinical trial of patients with high-risk features of PA found that the seated saline suppression test had limited ability to identify lateralizing disease on adrenal vein sampling (AVS) (64.4% accuracy; 95% CI, 56.4–71.8), directly questioning its utility for triaging surgical candidates to lateralization testing [30]. Collectively, these findings support moving away from suppression testing as a universal diagnostic requirement and raise uncertainty about whether it provides sufficient incremental information to guide progression to AVS, even among patients with an intermediate probability of lateralizing disease.

### Imaging

Among patients with likely PA who are considering surgery, adrenal CT is used to characterize adrenal anatomy, identify concerning masses, and facilitate AVS and surgical planning. However, cross-sectional imaging cannot reliably establish functional laterality of PA. In a pooled analysis of 38 studies (n = 950), Kempers et al. reported that CT/MRI misclassified lateralization in 37.8% of cases, leading to inappropriate management in up to one-third of patients [31]. Consistent with these findings, Williams et al. reported that CT/MRI correctly lateralized disease in only 64% of surgically cured patients following AVS-guided management [32].

These limitations reflect a fundamental constraint: CT and MRI define adrenal anatomy but do not directly assess hormone secretion. Imaging cannot reliably distinguish aldosterone-producing adenomas from nonfunctioning adrenal nodules, which are increasingly prevalent with age. Apparent unilateral adenomas may therefore be incidental, and the true source of aldosterone excess may arise from the contralateral gland. Accordingly, adrenal imaging should not be used in isolation to determine functional lateralization in most patients.

### Lateralization Using Adrenal Vein Sampling

For patients with PA who are interested in and eligible for surgical treatment, the next step in evaluation is to assess for lateralizing aldosterone production. Decisions regarding further subtype evaluation should integrate the biochemical phenotype, surgical candidacy and comorbidities, patient preference, and available expertise and resources. AVS remains the principal test for functional localization. The likelihood of lateralizing disease generally increases with biochemical severity, including more marked aldosterone excess, renin suppression, and hypokalemia. AVS may be omitted when the subtype of disease is already established or when results are unlikely to alter management. In younger patients (typically < 35 years) with clinically severe PA, characterized by hypokalemia, suppressed renin, and substantially elevated aldosterone, and a unilateral adrenal adenoma (> 1 cm) on imaging, the likelihood of asymmetric disease is sufficiently high that adrenalectomy may be considered without prior AVS. AVS is also unnecessary when bilateral disease is already established or when surgery is not being considered [7].

Interpretation of AVS is a 2-step process. Technical validity is first established by confirming successful cannulation of both adrenal veins, typically using the selectivity index, defined as the ratio of cortisol concentrations in the adrenal veins to the inferior vena cava. Values above the established thresholds indicate reliable sampling; a threshold of 3 is frequently utilized. Lateralization is then assessed using cortisol-corrected aldosterone ratios. The lateralization index, calculated as the ratio of aldosterone-to-cortisol on the dominant side to that on the nondominant side, is used to distinguish asymmetric from bilateral aldosterone excess. A lateralization index > 4 is generally considered consistent with asymmetric or unilateral disease, whereas values < 3 suggest bilateral secretion. Intermediate values represent an indeterminate range and should be interpreted in conjunction with contralateral suppression, clinical features and imaging findings [7].

AVS is subject to both technical and biological variability and is highly operator-dependent [33]; success rates can exceed 90% in highly experienced centers [34]. Despite its invasive nature, complication rates are low; in a multicenter international study of 2604 procedures, adrenal vein rupture occurred in 0.61% of cases, with no mortality and all complications managed conservatively [35]. To improve technical success, cosyntropin is often administered during AVS to stabilize cortisol secretion and enhance the confirmation of cannulation. However, its use remains debated. Although cosyntropin improves cannulation reliability, it may alter lateralization and therefore the classification of disease subtypes, particularly in borderline cases. Yozamp et al. reported discordance between pre- and post-cosyntropin measurements in approximately 19% of cases [36]. Thus, AVS remains the reference standard for functional lateralization, but its interpretation depends on procedural protocol, technical expertise, and the clinical phenotype.

### Emerging Molecular Imaging

Recent advances in nuclear imaging have introduced positron emission tomography (PET)-based approaches for subtype evaluation in PA, including modalities targeting aldosterone synthase (CYP11B2) or cell-surface receptors such as CXCR4 expressed in aldosterone-producing tissue. These techniques aim to determine the functional localization of aldosterone secretion and distinguish asymmetric or unilateral disease from bilateral disease. Current guidance emphasizes the potential for these approaches to reduce the reliance on AVS by enabling noninvasive, functionally specific imaging for subtype classification. This reflects recognition that AVS, although the current reference standard, is invasive, technically demanding, and dependent on specialized expertise.

In clinical studies, these modalities have demonstrated promising performance. In a prospective within-patient study of 169 patients who underwent both post-dexamethasone ^11C-metomidate PET/CT and AVS, molecular imaging was noninferior to AVS for predicting surgical outcomes [37]. In a prospective cohort of 197 participants, Zheng et al. reported that ^68Ga-pentixafor PET/CT-guided vs. AVS-guided surgical management was associated with comparable postoperative clinical and biochemical outcomes [38]. In a separate retrospective study, Zuo et al. reported 71.3% concordance in subtype classification between CXCR4 PET imaging and AVS among the 136 patients who completed both investigations [39]. Thus, functional imaging may directly localize active adrenal lesions, rather than relying on indirect sampling, while achieving performance broadly comparable to that of AVS. These modalities offer a noninvasive approach that may reduce reliance on AVS. However, these modalities remain available primarily in specialized centers and for research purposes; further validation, standardization, and regulatory development will be needed before routine use.

## Management of Primary Aldosteronism

### Comparative Outcomes of Surgical and Medical Treatment

Management of PA is directed at reducing aldosterone excess and its associated cardiovascular risk. Treatment options include adrenalectomy or lifelong medical therapy. Current guidelines recommend selecting between these approaches based on disease subtype, with surgery typically offered to patients with asymmetric disease and medical therapy used for bilateral disease or when surgery is not preferred or feasible. Observational studies and meta-analyses associate adrenalectomy for lateralizing PA with more favorable blood pressure and cardiovascular outcomes than MRA-treated cohorts, although these comparisons are susceptible to confounding by disease subtype, surgical selection, and adequacy of medical therapy [40, 41]. Additionally, outcomes with medical therapy may depend on the adequacy of the biochemical response. Hundemer et al. demonstrated that patients with persistently suppressed renin levels despite medical treatment had a 2.5-fold higher risk of atrial fibrillation than patients with essential hypertension [42]. In contrast, patients in whom MRA therapy resulted in unsuppressed renin had no excess risk of atrial fibrillation relative to patients with primary hypertension, similar to surgically treated patients. These findings highlight how differences in outcomes may reflect not only treatment modality but also whether renin suppression is reversed. For medically treated patients with uncontrolled hypertension and persistently suppressed renin, current guidance supports intensifying medical therapy to achieve a rise in renin. It may be beneficial to prioritize MRAs over other antihypertensive agents in order to achieve unsuppressed renin and blood pressure control, but it is less clear whether renin should be targeted when blood pressure is already controlled.

### Surgery

Adrenalectomy is the treatment of choice for patients with asymmetric or unilateral PA who are appropriate surgical candidates, with the goal of eliminating excess aldosterone production and reducing associated cardiovascular risk. Minimally invasive adrenalectomy, using laparoscopic or robot-assisted techniques, is the standard of care and may be performed via anterior (transabdominal) or posterior (retroperitoneal) approaches [43, 44]. Complete biochemical success is achieved in the great majority of appropriately selected patients, although complete resolution of hypertension is less common. In the international Primary Aldosteronism Surgical Outcome (PASO) cohort, 94% achieved complete biochemical success (defined as correction of hypokalemia and normalization of the aldosterone-to-renin ratio), whereas 37% achieved complete clinical success (defined as normotension without antihypertensive medications) and an additional 47% achieved partial clinical success (defined as lower blood pressure and/or reduced antihypertensive medication requirements) [45]. Thus, persistent hypertension after surgery does not necessarily indicate persistent PA, but may instead represent concomitant primary hypertension [46–48]. MRA therapy can be safely continued preoperatively, particularly if needed for sufficient blood pressure control [49]. Following surgery, patients should undergo reassessment to confirm biochemical resolution and guide ongoing management.

### Steroidal Mineralocorticoid Receptor Antagonist Therapy

Steroidal MRAs, primarily spironolactone and eplerenone, constitute the cornerstone of medical therapy for PA, particularly in patients with bilateral disease or when surgery is not pursued. The goal of therapy is to antagonize aldosterone-mediated mineralocorticoid receptor activation and thereby reduce blood pressure, correct hypokalemia, and mitigate cardiovascular risk. Current guidelines support MRA therapy as a standard treatment, with spironolactone generally used as a first-line therapy because of its potency and cost, and eplerenone used as an alternative when adverse effects limit tolerability.

Outcomes with medical therapy depend in part on the adequacy of the biochemical response. In a meta-analysis of 6621 patients treated with MRAs, Katsuragawa et al. reported that patients with unsuppressed posttreatment renin had a significantly lower risk of cardiovascular events than those with persistent renin suppression did (hazard ratio, 0.43) and were more likely to achieve lower blood pressure [50].

Differences between available MRAs influence the ability to achieve effective receptor blockade while maintaining tolerability. Spironolactone may provide more effective control of hypokalemia and reduce antihypertensive medication burden compared with eplerenone, although overall blood pressure reductions are broadly similar across agents [40]. However, spironolactone is associated with a higher frequency of adverse antiandrogen-related effects, including gynecomastia and menstrual irregularities, whereas eplerenone is better tolerated but may require higher and twice daily dosing to achieve comparable receptor blockade. Dose selection further affects treatment tolerability and effectiveness. In a retrospective cohort of 394 patients with PA, Sconfienza et al. reported no significant difference between low-dose spironolactone (≤ 50 mg per day) and high-dose therapy (> 50 mg per day) in terms of blood pressure control, potassium levels, renin levels, or cardiovascular events. However, adverse effects were more frequent with higher doses, particularly in men, and treatment discontinuation occurred more often in the high-dose group [51].

Thus, MRA therapy should be individualized and actively titrated according to blood pressure, potassium level, renal function, and the renin response to achieve effective receptor blockade. A rise in renin level may serve as an additional marker of adequate treatment, particularly when hypertension remains uncontrolled. Dose selection and agent choice should balance efficacy with tolerability, with adjustment or substitution when adverse effects occur.

### Nonsteroidal Mineralocorticoid Receptor Antagonist Therapy

The principal pharmacologic advantage of nonsteroidal MRAs is greater receptor selectivity than steroidal MRAs and fewer androgen- and progesterone-mediated adverse effects, although hyperkalemia remains an important safety consideration [52]. Evidence for the use of nonsteroidal MRAs in patients with PA is now emerging [53, 54]. In a 2026 multicenter prospective study of 57 patients with PA, 12 weeks of finerenone was associated with 7 mm Hg lower daytime ambulatory systolic blood pressure from baseline, an increase in the proportion of patients with normal serum potassium, and a mean increase in plasma renin activity [54]. However, the study was small and single-arm, and randomized head-to-head comparisons with spironolactone or eplerenone are needed to establish whether there is a role for finerenone in PA.

### ENaC Inhibitors

Epithelial sodium channel (ENaC) inhibitors, including amiloride and triamterene, are alternative medical therapies for PA that may be used when MRAs are not tolerated or are contraindicated. They reduce sodium reabsorption in the distal nephron downstream of aldosterone signaling, thereby reversing sodium retention and lowering blood pressure. Direct evidence for ENaC inhibitors in PA is limited: head-to-head trials comparing ENaC inhibitors with MRAs in PA are lacking and comparative data derives largely from patients with low-renin and resistant hypertension. Indirect evidence from patients with resistant and low-renin hypertension suggests that amiloride can result in blood pressure reductions similar to those achieved with spironolactone. In the SALT crossover trial, amiloride (40 mg) lowered blood pressure to a degree comparable to that of spironolactone and thiazide diuretics in patients with low-renin hypertension and an elevated ARR [55]. Similarly, in PATHWAY-2, amiloride demonstrated antihypertensive efficacy comparable to that of spironolactone in patients with resistant hypertension [56]. However, ENaC inhibitors do not directly block mineralocorticoid receptor activation, and whether they provide the same protection against aldosterone-mediated organ injury as MRAs has not been established. Current guidelines therefore recommend MRAs as first-line medical therapy, with ENaC inhibitors considered second-line or substitute therapy when MRAs are not tolerated.

### Future Potential for Aldosterone Synthase Inhibitors

Aldosterone synthase inhibitors (ASIs), including baxdrostat and lorundrostat, represent an emerging class of therapies that directly reduce aldosterone production. ASIs are designed to selectively inhibit aldosterone synthesis (CYP11B2) while largely preserving cortisol production, reducing aldosterone at its source rather than blocking the receptor. This mechanism may offer a complementary or alternative approach to achieve effective suppression of aldosterone activity. These agents are not yet included in clinical guidelines and remain under investigation for PA. Phase 3 trials have now demonstrated clinically meaningful (i.e., 9–10 mmHg placebo-corrected) blood pressure reductions with both lorundrostat and baxdrostat in uncontrolled or resistant hypertension [57, 58]. Baxdrostat was approved by the U.S. Food and Drug Administration in 2026 as add-on therapy for adults with hypertension inadequately controlled on other agents [59]. Evidence specific to PA remains limited, and long-term effects of ASIs on cardiovascular and renal outcomes are unknown. In a randomized phase 2 trial of 35 patients with PA, dexfadrostat phosphate reduced the median ARR from 15.3 to 0.6 (a 92% relative reduction) and lowered 24-h ambulatory systolic blood pressure by 11 mm Hg over the treatment period (both p < 0.0001) [60]. In a phase 2 a study of 15 patients with PA, baxdrostat resolved or reduced hypertension, suppressed the ARR (often to a degree comparable with adrenalectomy), and corrected hypokalemia [61]; a phase 3 trial in PA is ongoing [62]. Across ASI trials, hyperkalemia and a reduction in estimated glomerular filtration rate have been observed and require monitoring. ASIs may expand future treatment options for PA, but their role in clinical practice depends on the confirmation of their long-term efficacy, safety, and comparative effectiveness relative to those of established therapies.

## Implementation

Despite these advances in the diagnosis and treatment of PA, the dominant gap remains implementation. Contemporary guidelines recommend broader screening for PA, yet implementation remains limited, with fewer than 3% of eligible patients undergoing testing. Many patients are not screened because PA continues to be perceived as uncommon in routine practice, despite its relatively high prevalence. Screening is often reserved for patients with hypokalemia, although most patients with PA are eukalemic. In addition, clinicians frequently view testing as complex, particularly with respect to medication effects on renin and aldosterone and interpretation of the ARR. Persistent misconceptions across the PA evaluation and management pathway may further impede implementation (Table 1). Consequently, screening is most commonly performed by subspecialists such as endocrinologists and nephrologists, rather than more opportunistically in primary care [15].

**Table 1:** Common misconceptions in primary aldosteronism evaluation and management

Common Misconception	Reality (What Current Evidence and Guidelines Support)
PA screening is only indicated in patients with resistant hypertension	Screening is recommended well beyond resistant hypertension. Contemporary guidelines extend screening to stage 2 hypertension and multiple high-risk phenotypes, including hypokalemia, adrenal incidentaloma, obstructive sleep apnea, early-onset hypertension or stroke, and family history of PA
PA only occurs in patients with hypokalemia	Most patients with PA are eukalemic. Hypokalemia is present in a minority of cases and is more common in severe or asymmetric disease. Absence of hypokalemia does not exclude PA
Renin and aldosterone must be measured under strict positioning conditions	Morning measurement with the patient seated is generally adequate for initial screening; elaborate positioning protocols are not required
All antihypertensive medications must be stopped prior to screening	Initial screening can generally be performed without medication withdrawal. When results are negative or equivocal despite high clinical suspicion, selective withdrawal of medications that can obscure renin suppression may improve interpretability when safe and feasible
A single ARR is sufficient to rule out PA	ARR results should be interpreted together with aldosterone, renin, medications, assay characteristics, and pretest probability. Repeat testing is particularly useful when results are borderline or discordant with the clinical phenotype
All patients require confirmatory testing to diagnose PA	Repeat ARR is not needed to confirm a positive test. Aldosterone suppression testing is now used selectively rather than universally. However, its incremental utility remains uncertain, including for identifying patients likely to have lateralizing disease, and it may be omitted when results are unlikely to change next steps in evaluation or management
PA is primarily caused by unilateral adrenal adenomas	PA is heterogeneous and includes both lateralizing and nonlateralizing disease; nonlateralizing/bilateral disease is particularly common in milder phenotypes
A unilateral adrenal nodule on CT identifies the source of aldosterone excess	Cross-sectional imaging defines adrenal anatomy but cannot reliably determine functional laterality. Nonfunctioning adrenal nodules are common, and AVS remains the reference standard for lateralization in most surgical candidates
Successful adrenalectomy will cure hypertension	Biochemical cure after adrenalectomy is substantially more common than complete resolution of hypertension. Persistent hypertension after successful surgery may reflect coexisting primary hypertension and does not necessarily indicate persistent PA

Abbreviations: *ARR* aldosterone-to-renin ratio; *AVS* adrenal vein sampling; *MRA* mineralocorticoid receptor antagonist; *PA* primary aldosteronism

Interventions that integrate screening into clinical workflows have been developed to address these barriers. In a quality improvement study, Charoensri et al. evaluated a noninterruptive best-practice advisory embedded within the electronic health record that identified patients meeting screening criteria and prompted consideration of testing. The screening rate increased to 14% of eligible patients, with 70.5% completing testing and 17.4% demonstrating positive results [63]. In a pilot study, Passman et al. implemented an electronic health record–based intervention that combined automated patient identification with an active-choice prompt requiring clinicians to accept or decline screening. Screening increased to 16.4% in intervention clinics, compared with 1.8% in control clinics [64]. Improving implementation may also require strategies that standardize interpretation of screening tests. The integration of clinical decision support within electronic health records represents one such approach. These systems can incorporate medication use, assay characteristics, and clinical context into result interpretation. Early studies suggest that such approaches may increase screening uptake and streamline diagnostic pathways [65]. Standardized interpretive comments within laboratory reports may improve context-specific guidance, particularly in nonspecialist settings. For example, an elevated ARR may be accompanied by a comment indicating that renin-suppressing medications can lead to false-positive findings and should be considered in interpretation.

## Conclusions and Future Directions

Primary aldosteronism is increasingly recognized as a common and heterogeneous form of hypertension in which delayed diagnosis may prolong exposure to aldosterone-mediated cardiovascular and kidney injury. Recent guidelines translate this recognition into broader screening and more pragmatic ARR testing, while shifting downstream evaluation toward a treatment-directed strategy. Patients who do not pursue surgery can often begin MRA therapy without an exhaustive localization workup, whereas surgical candidates should undergo targeted evaluation for lateralizing disease, typically adrenal imaging and AVS, with selective use of aldosterone suppression testing. For medically treated patients, normalization of blood pressure and potassium are the primary goals, with a rise in renin providing an additional marker of adequate mineralocorticoid receptor blockade when hypertension remains uncontrolled. Molecular imaging, nonsteroidal MRAs, and ASIs may further expand diagnostic and therapeutic options, but comparative and long-term outcome data are needed. The principal challenge is now to translate these advances into routine hypertension care through scalable strategies for case identification, test interpretation, referral, and targeted treatment.

## Key References

- Leung AA, Padwal RS, Hundemer GL, Venos E, Campbell DJT, Holmes DT, et al. Seated Saline Suppression Test for Lateralizing Primary Aldosteronism. Hypertension. 2026;83(5):e26008.
  - ○ Blinded, prospective study that demonstrated the limited ability of saline suppression testing to identify lateralizing PA, questioning its utility for determining which surgical candidates should proceed to AVS. This is particularly important because it challenges even the more selective role retained for suppression testing in current guidelines.
- Adler GK, Stowasser M, Correa RR, Khan N, Kline G, McGowan MJ, et al. Primary Aldosteronism: An Endocrine Society Clinical Practice Guideline. J Clin Endocrinol Metab. 2025;110(9):2453–95.
  - ○ Major contemporary guideline broadening PA screening and supporting more pragmatic ARR interpretation, selective suppression testing, and treatment-directed evaluation.
- Goupil R, Desbiens LC, Merabtine A, Agharazii M, Madore F, Vaidya A, et al. Subclinical Primary Aldosteronism and Major Adverse Cardiovascular Events: A Longitudinal Population-Based Cohort Study. Circulation. 2025;152(13):913–23.
  - ○ Demonstrated cardiovascular risk across the spectrum of renin-independent aldosterone production, supporting recognition of PA as a continuum extending below conventional diagnostic thresholds.
- Goodchild E, Wu X, Senanayake R, MacFarlane J, Argentesi G, Laycock K, et al. Molecular Imaging Versus Adrenal Vein Sampling for the Detection of Surgically Curable Primary Aldosteronism: A Prospective Within-Patient Trial. Annals of internal medicine. 2025;178(3):336–47.
  - ○ Prospective within-patient study demonstrating that molecular imaging can provide clinically meaningful localization of surgically curable PA, supporting its potential as a noninvasive alternative to AVS.
- Katsuragawa S, Le MV, Fuller PJ, Yang J. Post-treatment renin status and cardiovascular, renal, and mortality outcomes in medically treated primary aldosteronism: a systematic review and meta-analysis. Lancet Diabetes Endocrinol. 2025;13(12):1041–53.
  - ○ Meta-analysis linking post-treatment renin unsuppression with lower cardiovascular risk, strengthening the evidence for renin response as a marker of adequacy of medical therapy.
- Turcu AF, Freeman MW, Bancos I, Ben-Shlomo A, Hamidi O, Hamrahian AH, et al. Phase 2 a Study of Baxdrostat in Primary Aldosteronism. The New England journal of medicine. 2025;393(5):515–8.
  - ○ Provided early PA-specific evidence that ASIs can improve blood pressure and biochemical manifestations of PA, supporting a potentially important new therapeutic approach.

## Data Availability

No datasets were generated or analysed during the current study.
